# Changes in mental health levels among Chinese athletes from 1995 to 2023

**DOI:** 10.3389/fpsyg.2024.1343522

**Published:** 2024-03-20

**Authors:** Yue Xi, Fengbo Liu, Jian Yang

**Affiliations:** ^1^Key Laboratory of Adolescent Health Assessment and Exercise Intervention of Ministry of Education, East China Normal University, Shanghai, China; ^2^College of Physical Education and Health, East China Normal University, Shanghai, China; ^3^School of Physical Education, Zhengzhou University of Light Industry, Zhengzhou, Henan, China

**Keywords:** athletes, SCL-90, mental health, cross-temporal meta analysis, meta-analysis

## Abstract

**Objective:**

In recent years, with the increasing intensity of sports competition and social pressure, the issue of mental health among athletes have gradually attracted attention. Understanding the changing trends of athletes’ mental health is of great significance for formulating effective intervention measures and safeguarding the mental health of athletes.

**Methods:**

A total of 306 articles were accumulated from September to November 2023 by selecting literature from databases that measured the mental health of Chinese athletes using the Symptom Checklist-90 (SCL-90). Based on the screening criteria, 28 articles (*N* = 4,227) were finally included. A cross-sectional historical meta-analysis of these 28 studies using the SCL-90 between 1995 and 2023 was conducted. Based on cross-sectional historical meta-analysis methods, specific statistical methods, such as correlation and regression analyses, were used to examine trends over time in the scores of the nine SCL-90 factors measured by the athletes in the studies, as well as athlete type and gender differences.

**Results:**

(1) Athletes’ scores on the depression, hostility, phobic anxiety, and paranoid ideation factors gradually increased with the change of era, and the explanation rate of the variation of each factor by era ranged from 18.8 to 27.2%; (2) During the period from 1995 to 2023, the mental health of athletes in China gradually deteriorated on the factors of depression, hostility, and phobic anxiety, the rate of change was rapid, with a medium to large magnitude (0.28–0.42); (3) The scores of female athletes on the depression and psychoticism factors scores showed a significant upward trend, while male mental health scores for each factor did not improve significantly over the years; (4) College athletes’ scores on the depression, anxiety, hostility, phobic anxiety, paranoid ideation, and psychoticism factors showed an upward trend as the years changed.

**Conclusion:**

Chinese athletes’ mental health has been on a downward trend for the past 28 years, which needs to be a cause for concern.

## 1 Introduction

Athletes are one of the most important subjects of competitive sports. They are subjected to high-stress training and competition tasks, as well as other social activities, recovery from physical injuries, and the scrutiny and pressure of public media. They belong to the high stress group, and a series of problems will occur when the stressor exceeds their coping ability. In recent years, the International Athletes Forum (IAF) has placed greater emphasis on the issue of mental health in athletes, as well as strong references to mental health in Olympic Agenda 2020 + 5, the IOC and the Olympic Movement’s strategic road map to 2025 (Schinke et al., 2017; Moesch et al., 2018; Reardon et al., 2019). Mental health is one of the core elements of an athlete and an important resource before and after the entire athletic career (Moesch et al., 2018).

China’s earliest study on athletes’ mental health was in 1993. Tan et al. (1993) analyzed the universality and particularity of athletes’ mental health problems based on Vitaliano’s theoretical model, and since then China has started to conduct systematic research on athletes’ mental health. In the domestic research, the results of the research on the mental health of athletes are not uniform. The scores of some professional athletes in Jiangsu Province in the six factors (somatization, compulsion, anxiety, hostility, paranoia, psychosis) are significantly higher than the domestic youth norm (Shen, 2007), and studies have shown that most high-level athletes in the obsessive symptoms, interpersonal sensitivity, anxiety, hostility and other factors are significantly higher than the national norm (Tan et al., 2010, 2014).Studies have also shown that the mental health of Chinese athletes is good, for example, the compulsive symptoms, interpersonal sensitivity, depression and other factors of juvenile track and field athletes are better than that of middle school students (Zhang, 2003). The mental health level of college athletes is significantly higher than that of ordinary college students, and the health level of compulsion, interpersonal, depression, paranoia and other factors is also significantly higher than the national norm level (Song et al., 1997).In addition to obsessive symptoms and psychotic symptoms, the other factors of elite shooters were lower than the national normal adult norm (Chen and Lu, 2014). Shi et al. (2020) argues that this is partly a reflection of the fact that researchers may have different definitions and understandings of mental health in elite athletes, and may also affect the diagnosis of subsequent mental health problems. As time goes by, the importance of research on athletes’ mental health becomes increasingly prominent. Particularly, following the outbreak of the COVID-19 pandemic, athletes are facing unprecedented challenges, with their sleep, diet, physical activities, and mental health being significantly affected (Taheri et al., 2023). Both elite athletes and amateur athletes have been affected by factors such as stress, lockdown measures, interruptions in training and competition schedules during the pandemic. These have led to negative effects on athletes’ mental health to some extent, such as depression and anxiety (Taheri et al., 2023). Therefore, through a longitudinal examination of historical research, we can better grasp the developmental trends of athletes’ mental health, providing essential references for designing more effective interventions and support measures in the future.

In the existing researches on athletes’ mental health, most of them are conducted in specific time and place, and the overall longitudinal studies are few. Qi et al. (2003) conducted a follow-up survey on the mental health of college students in physical education colleges in 2003 and found that the mental health level of college students had declined during the 4 years in college. No other domestic studies tracking the mental health of athletes or college athletes have been found. The current studies are based on small sample studies conducted in specific time and specific region, so there may be inconsistency in the research results. At present, the most effective solution is the cross-sectional historical meta-analysis method, which can effectively analyze the changing characteristics of athletes’ mental health by studying the psychological differences or variations related to a large span of time and times (Xin and Zang et al., 2009; Xin et al., 2012; Yi et al., 2012). Therefore, whether the mental health status of athletes has a downward trend year by year is one of the contents of this study. At the same time, there is a lack of research on the differences between different types of athletes and different gender athletes. Therefore, this study examines whether there are differences in the mental health level of athletes in different subgroups through general meta-analysis, which is the second content of this study. Subgroup athletes are divided into two categories: professional athletes and college student athletes. A professional athlete refers to individuals who engage in sports as a profession, participating in various professional competitions to earn rewards and compensation. A college student athlete, on the other hand, is a student pursuing university studies who utilizes the platform provided by the university to participate in various competitions to enhance their physical fitness and skills. College student athletes are often students of sports science or related fields and are distinct from professional athletes.

To sum up, this study adopts cross-sectional historical meta-analysis research method to collect research reports of Chinese athletes using SCL-90 scale from 1995 to 2023 to explore and analyze the changing trend, speed and specific factors of the overall mental health status of Chinese athletes over the years. Understanding these dynamics is essential for developing targeted interventions and support systems to enhance the mental health of athletes in China. Moreover, this study seeks to investigate the gender-based variations in mental health trajectories among athletes and delve into the distinct patterns observed across different types of athletes. By delving into these nuances, we can gain a comprehensive understanding of the unique challenges and needs faced by various athlete groups, paving the way for tailored mental health strategies and interventions within the Chinese sporting community. Ultimately, this study is pivotal in informing evidence-based strategies to promote the mental well-being of Chinese athletes and foster a healthier and more supportive sports environment.

## 2 Methods

### 2.1 Literature search

The SCL-90 is an instrument to estimate psychological problems with a five-point rating scale, ranging from 1 (not at all) to 5 (extremely). It has a wide coverage of various mental health disorders and contains nine subscales about mental health (i.e., somatic complaints, obsessive-compulsive symptoms, interpersonal sensitivity, depression, anxiety, hostility, phobic anxiety, paranoid ideation, and psychoticism), with a higher score suggesting a lower mental health level (Derogatis et al., 1973; Xin et al., 2019). The SCL-90 scale has a wide range of application and can accurately assess the degree of subjective disease perception and distribution characteristics (Wang, 1984). In addition, the Chinese version of the SCL-90 is the most frequently used tool for measuring Chinese athletes’ mental health levels (Liu et al., 2002; Tang et al., 2013). Thus, this study collected data from original research on the SCL-90 scores of Chinese athletes to conduct a cross-temporal meta-analysis.

The sources of literature for the current research were three academic literature databases in Chinese: CNKI, Wanfang, Chongqing VIP Information. These databases are frequently used in China and include Chinese journals of sciences and social sciences published after 1985, as well as master’s theses and doctoral dissertations. We used search terms such as “SCL-90,” “athletes,” “college athletes,” “psychological problems,” and “mental health.” Additionally, we searched five academic literature databases in English, including Elsevier, Wiley, ProQuest, PubMed, and Web of Science, using the same keywords. Only articles that provided the average SCL-90 score, sample size, and publication year were collected. In addition, the year of data collection was coded as 2 years before the publication of the previous study, unless a specific year was mentioned in the article (Twenge, 2001; Xin and Chi, 2008; Xin et al., 2019).

### 2.2 Inclusion rules

**Figure 1:**
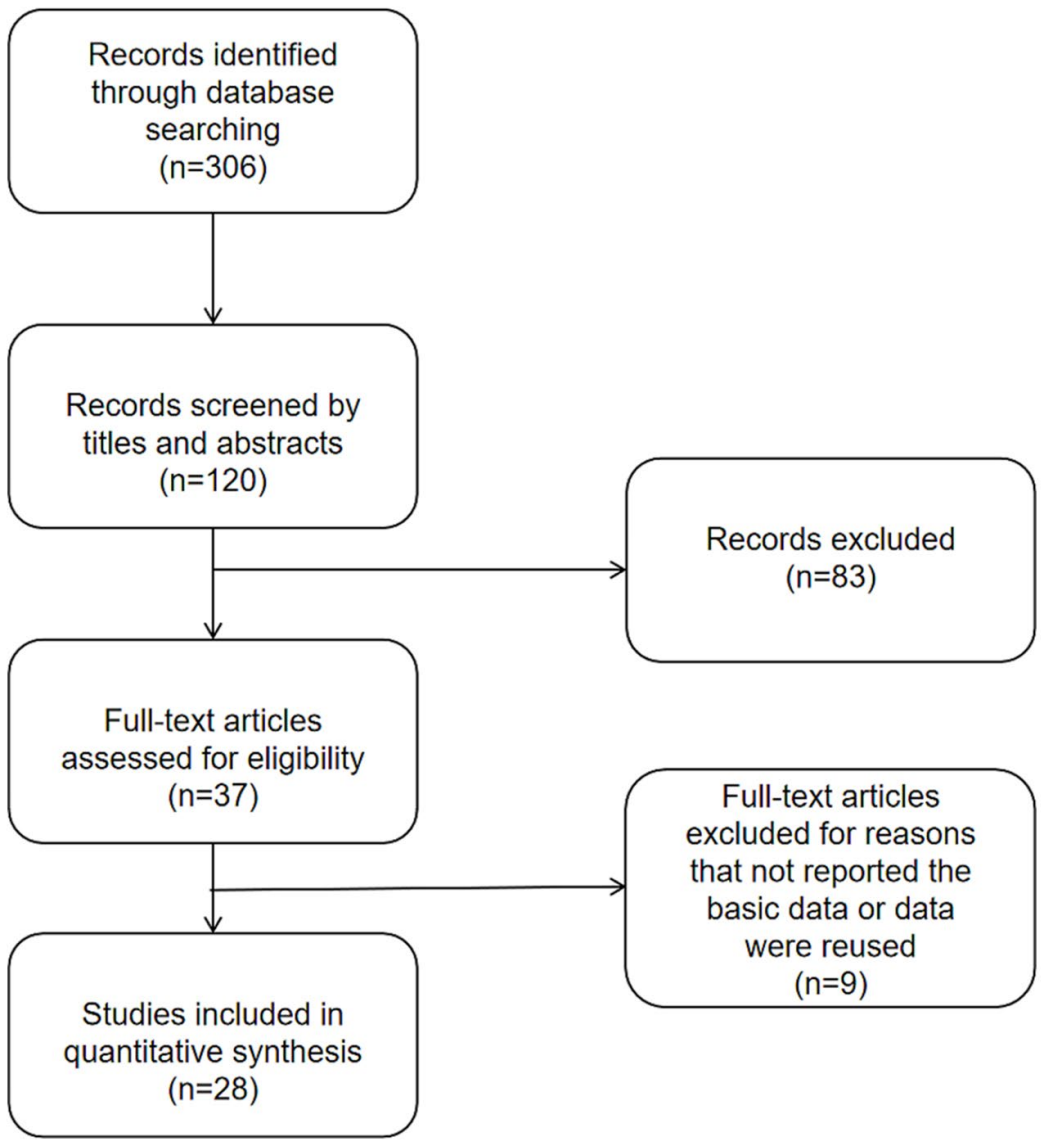
PRISMA flow chart for the screening of studies.

Studies that met the following special inclusion rules were included in the current cross-temporal meta-analysis: (1) studies using the SCL-90 as the tool; (2) studies reporting mean scores and sample sizes for the total or unselected subgroups; (3) studies in which the participants were Chinese athletes; (4) studies in which all participants were from mainland China; (5) studies published before September 2023; and (6) for studies in which the same data were published twice or more, the earliest study was selected. To illustrate the inclusion strategy and the results of the search strategy, a PRISMA diagram is depicted in Figure 1.

As shown in Figure 1 and Table 1, the final sample included 28 studies involving 4,227 athletes between 1995 and 2023 (the year of data collection). No English literature met the inclusion criteria. Detailed information for the studies included in the meta-analysis can be found in Table 1.

**Table 1:** Description of studies included in the cross-temporal meta-analysis.

First authors	Year of data collection	*N*	Publication class	Types of athletes	Somatic complaints	Obsessive compulsive	Interpersonal sensitivity	Depression	Anxiety	Hostility	Phobic anxiety	Paranoid ideation	Psychoticism
Shu	2022	40	3	2	1.17 ± 0.15	1.58 ± 0.27	1.43 ± 0.33	1.25 ± 0.21	1.26 ± 0.21	1.27 ± 0.26	1.21 ± 0.26	1.27 ± 0.27	1.34 ± 0.2
Zhao	2019	173	3	2	1.66 ± 0.66	1.92 ± 0.74	1.74 ± 0.69	1.67 ± 0.74	1.62 ± 0.71	1.67 ± 0.69	1.52 ± 0.7	1.69 ± 0.69	1.56 ± 0.69
Lv	2017	12	1	1	1.62 ± 0.31	2 ± 0.37	1.73 ± 0.34	1.69 ± 0.36	1.5 ± 0.27	2.2 ± 0.81	1.4 ± 0.38	1.62 ± 0.61	1.6 ± 0.28
Tan	2014	359	2	1	1.38 ± 0.62	1.75 ± 0.65	1.78 ± 0.63	1.56 ± 0.67	1.49 ± 0.64	1.75 ± 0.73	1.26 ± 0.57	1.47 ± 0.6	1.26 ± 0.59
Ding	2014	20	2	1	1.54 ± 0.43	1.95 ± 0.58	1.54 ± 0.52	1.52 ± 0.39	1.54 ± 0.65	1.62 ± 0.5	1.41 ± 0.57	1.47 ± 0.33	1.53 ± 0.36
Sun	2011	376	2	2	1.52 ± 0.21	1.81 ± 0.26	1.92 ± 0.18	1.62 ± 0.18	1.71 ± 0.21	1.76 ± 0.46	1.42 ± 0.25	1.71 ± 0.31	1.49 ± 0.28
Ying	2010	195	1	1	1.59 ± 0.48	1.75 ± 0.54	1.61 ± 0.57	1.57 ± 0.5	1.46 ± 0.37	1.57 ± 0.59	1.38 ± 0.45	1.56 ± 0.56	1.41 ± 0.41
Niu	2009	404	1	2	1.53 ± 0.22	1.8 ± 0.25	1.93 ± 0.19	1.63 ± 0.19	1.7 ± 0.2	1.77 ± 0.47	1.43 ± 0.26	1.72 ± 0.32	1.48 ± 0.27
Zhen	2008	496	2	2	1.75 ± 0.64	2.03 ± 0.68	1.8 ± 0.65	1.82 ± 0.63	1.64 ± 0.67	1.69 ± 0.65	1.49 ± 0.55	1.71 ± 0.66	1.59 ± 0.53
Wei	2008	139	1	2	1.28 ± 0.31	1.54 ± 0.43	1.45 ± 0.41	1.4 ± 0.49	1.28 ± 0.36	1.39 ± 0.4	1.2 ± 0.34	1.42 ± 0.42	1.29 ± 0.33
Shen	2007	205	3	1	1.58 ± 0.45	1.83 ± 0.48	1.68 ± 0.61	1.54 ± 0.45	1.51 ± 0.46	1.77 ± 0.69	1.36 ± 0.49	1.61 ± 0.52	1.44 ± 0.4
Tan	2007	98	1	2	1.5 ± 0.48	1.67 ± 0.56	1.6 ± 0.61	1.51 ± 0.56	1.47 ± 0.51	1.57 ± 0.6	1.43 ± 0.57	1.52 ± 0.52	1.43 ± 0.5
Liu	2006	195	2	1	1.59 ± 0.48	1.75 ± 0.54	1.61 ± 0.57	1.57 ± 0.5	1.46 ± 0.37	1.57 ± 0.59	1.38 ± 0.45	1.56 ± 0.56	1.41 ± 0.41
Sun	2006	65	1	2	1.33 ± 0.26	1.39 ± 0.37	1.11 ± 0.37	1.06 ± 0.41	1.06 ± 0.33	0.94 ± 0.32	0.91 ± 0.28	0.87 ± 0.37	0.93 ± 0.21
Wu	2006	70	1	2	1.33 ± 0.34	1.47 ± 0.34	1.08 ± 0.4	1.1 ± 0.41	1.05 ± 0.21	1.08 ± 0.46	1 ± 0.41	0.95 ± 0.41	1.05 ± 0.34
ZHou	2005	139	2	2	1.28 ± 0.31	1.54 ± 0.43	1.45 ± 0.41	1.4 ± 0.49	1.28 ± 0.36	1.39 ± 0.4	1.2 ± 0.34	1.42 ± 0.42	1.29 ± 0.33
Liu	2005	205	2	1	1.58 ± 0.45	1.83 ± 0.48	1.68 ± 0.61	1.54 ± 0.45	1.51 ± 0.46	1.77 ± 0.69	1.36 ± 0.49	1.61 ± 0.52	1.44 ± 0.4
ZHou	2004	32	2	1	2.13 ± 0.616	2.49 ± 0.55	1.24 ± 0.53	2.14 ± 0.04	2.16 ± 0.049	2.27 ± 0.64	1.96 ± 0.6	2.3 ± 0.49	2.14 ± 0.51
Liu	2002	205	1	2	1.55 ± 0.43	1.45 ± 0.37	1.52 ± 0.43	1.46 ± 0.39	1.46 ± 0.35	1.49 ± 0.56	1.28 ± 0.33	1.42 ± 0.46	1.34 ± 0.41
CHen	2001	43	2	1	1.71 ± 0.55	1.67 ± 0.62	1.58 ± 0.61	1.55 ± 0.59	1.5 ± 0.56	1.5 ± 0.5	1.28 ± 0.37	1.49 ± 0.58	1.37 ± 0.47
Hu	2000	27	1	1	1.72 ± 0.34	1.81 ± 0.45	1.79 ± 0.47	1.77 ± 0.5	1.58 ± 0.43	1.67 ± 0.4	1.43 ± 0.45	1.79 ± 0.38	1.57 ± 0.4
ZHuo	2000	148	1	2	0.56 ± 0.44	1.93 ± 0.55	1.9 ± 0.55	0.74 ± 0.57	0.61 ± 0.48	1.61 ± 0.54	0.54 ± 0.54	0.72 ± 0.56	1.62 ± 0.44
ZHou	1998	64	1	1	0.75 ± 0.55	0.9 ± 0.59	0.81 ± 0.65	0.63 ± 0.55	0.57 ± 0.47	0.71 ± 0.63	0.54 ± 0.57	0.81 ± 0.61	0.5 ± 0.4
Tan	1997	131	2	2	1.57 ± 0.5	1.61 ± 0.5	1.55 ± 0.51	1.46 ± 0.44	1.44 ± 0.43	1.51 ± 0.57	1.3 ± 0.36	1.42 ± 0.45	1.36 ± 0.41
Qian	1997	141	1	2	1.54 ± 0.48	1.59 ± 0.49	1.51 ± 0.5	1.44 ± 0.43	1.43 ± 0.42	1.48 ± 0.55	1.29 ± 0.35	1.42 ± 0.46	1.35 ± 0.4
Song	1997	73	2	2	1.19 ± 0.63	1.56 ± 0.45	1.05 ± 0.55	1.33 ± 0.68	1.16 ± 0.48	1.41 ± 0.31	0.81 ± 0.62	0.95 ± 0.52	0.89 ± 0.52
CHen	1995	125	1	1	1.44 ± 0.36	1.65 ± 0.59	1.84 ± 0.53	1.41 ± 0.44	1.5 ± 0.39	1.59 ± 0.5	1.18 ± 0.31	1.41 ± 0.54	1.3 ± 0.42
Li	1995	47	2	2	1.31 ± 0.27	1.52 ± 0.48	1.08 ± 0.51	0.89 ± 0.43	1.04 ± 0.38	0.86 ± 0.45	0.88 ± 0.32	0.81 ± 0.49	0.92 ± 0.37

### 2.3 Coding of control variables

In the present study, publication class, sex ratio and types of athletes in every article were recorded as control variables in the cross-temporal meta-analysis because they may confound with the year. We coded the publication class was divided into three levels (Xin et al., 2019): first class (including the SSCI, SCI, CSSCI, and CSCI), second class (including publications from other sources) and third class (including master’s theses and doctoral dissertations). Publication class was controlled to avoid the effect of publication bias, which refers to the fact that studies with statistically significant results are more likely to be accepted for publication (Xin et al., 2019). And the types of athletes were divided into two levels: first class (including the professional athlete), second class (including college athletes). The sex ratio, an important variable for mental health scores, was also recorded in the database and controlled in regression analysis. Detailed information can be found in Table 2.

**Table 2:** Study variable coding tables.

Variable name	Code	Number of articles	*N*
Types of athletes	1 = Core journal	13	1,693
	2 = publication from other academic sources	12	2,116
	3 = dissertations and master’s theses	3	418
Gender	1 = male	8	1,148
	2 = female	9	1,032
Types of athletes	1 = professional athletes	13	1,482
	2 = college athletes	15	2,745

### 2.4 Data analysis strategy

First, for articles that only provided research data without comprehensive study results, the weighted statistics were obtained based on the provided data using the following formula (Li et al., 2022).


$$\bar{x}=\sum x_{i}n_{i}/\sum n_{i}$$

$$\begin{array}{l} S=\sum ns^{2}+\sum n{\left(x-\bar{x}\right)}^{2}/\sum n \end{array}$$

Where $\bar{x}$ , *S*, *n_i_*, *x_i_*, *x_i_*, *s_i_*, represent the average and standard deviation after synthesis, the sample size, average and standard deviation of a substudy, and analyze the changes of each factor in each stage. In addition, since most studies of SCL-90 used a 5-point scale ranging from 1 to 5, for the sake of uniformity, we converted the 3 studies with a score ranging from 0 to 4 into a score ranging from 1 to 5 by adding the mean of each factor by 1. To investigate the trend of mental health symptoms among athletes over different eras, a scatter plot will be created to illustrate the scores of each factor in the SCL-90 against the eras. Next, Pearson correlation analyses will be conducted between the scores of each factor in the SCL-90 and the eras. Furthermore, a weighted analysis will be performed to account for sample size, using eras as the independent variable and the scores of each factor in the SCL-90 as the dependent variables in a univariate linear regression analysis. This aims to examine the relationship between eras and mental health symptoms among college students.

In order to understand the specific amount of change in the mental health of different genders and athlete types Chinese athletes, according to previous researchers (Twenge et al., 2001, 2007), which is mainly measured by the effect size *d* or the explanation rate *r^2^*, and the formula for the two is as follows, where *SD* is the average standard deviation.


$${r_{i}}^{2}=\frac{d_{i}}{\sqrt{{d_{i}}^{2}+4}}$$

$$d_{i}=\frac{M_{2023i}-M_{1995i}}{SD_{i}}$$

This is achieved by establishing a regression equation (controlling sample size) that takes the mean value of each factor of SCL-90 as the dependent variable and the age as the independent variable: *y = Bx + C* (where *B* represents the unstandardized regression coefficient, *x* is the year, *C* is the constant term, and *y* is the average mental health quantity). After the regression equation of each factor is established, the mean scores of 1995 and 2023 *M_1995_* and *M_2023_* can be predicted. Following the usual practice of Twenge et al. (2007), the mean standard deviation in this study is obtained by averaging the standard deviations across all studies, unlike the calculation of the effect size in normal meta-analyses. This approach, which uses individual-level variables, effectively avoids the ecological fallacy. The statistical software used for this study is SPSS 25.0, and the reference management software used is EndNote 20.0.

## 3 Results

### 3.1 The changing trend of athletes’ mental health with time

**Figure 2:**
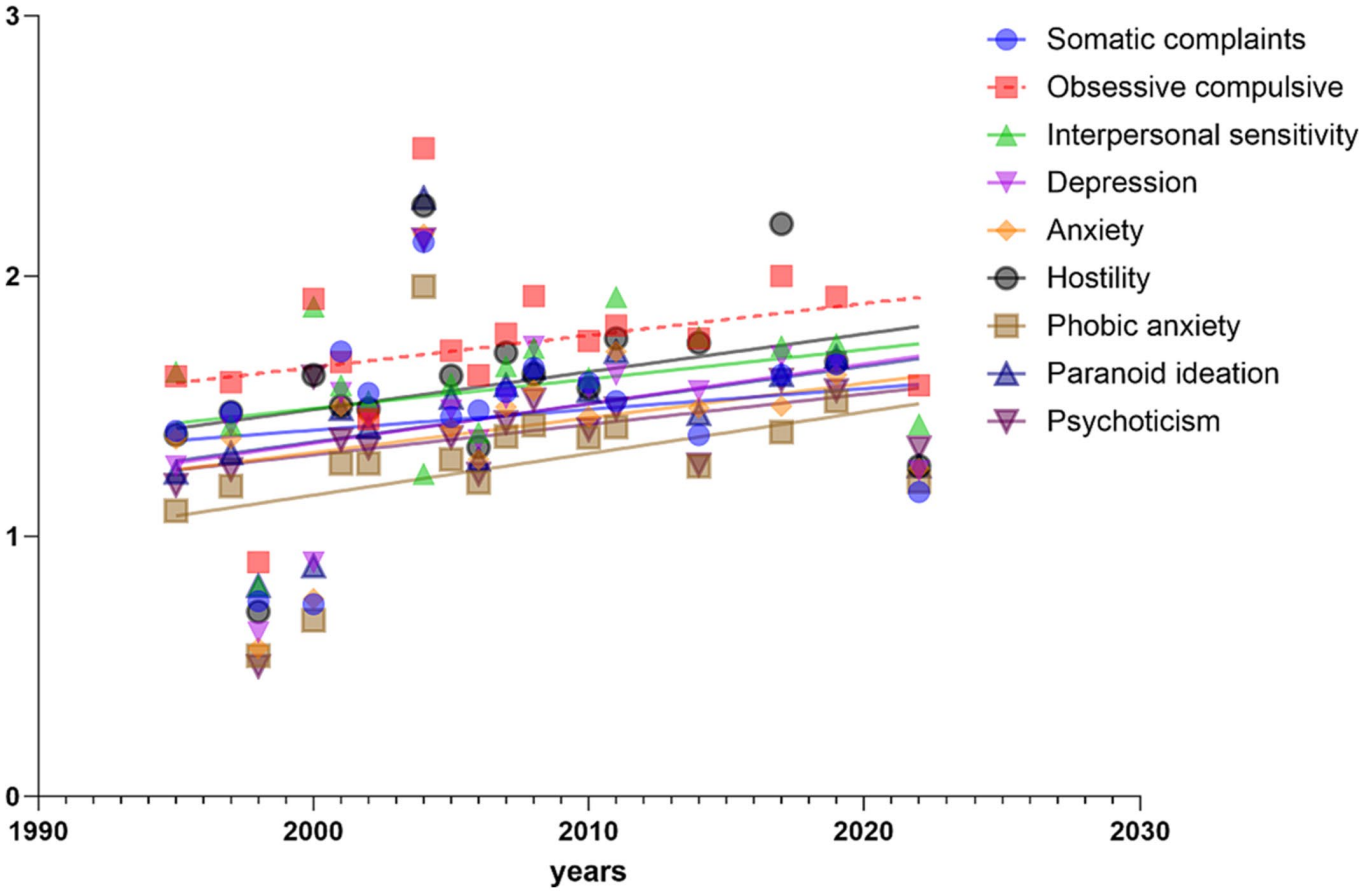
Correlations between the mean SCL-90 scores and year.

In this paper, by establishing the scatterplot of all factors and ages in SCL-90 scale, we can intuitively judge the change of athletes’ mental health in the process of age change. The scores of the 9 factors all improved to varying degrees with the change of years. See Figure 2 for the change of each factor with the change of years.

In order to explore whether the trend of change in the above scatter plot is significant, the correlation analysis of the 9 weighted factors in the SCL-90 scale is carried out. In this paper, the regression equation is established with age as the independent variable and scores of each factor as the dependent variable. Results As shown in Table 3 depression, hostility, phobic anxiety and paranoid ideation were significantly positively correlated with age (*p* < 0.05). The other factors were positively correlated with age, but none of them were significant. It can be seen that the age change can explain 25.6% of the variation of depression, 27.2% of the variation of hostility, 23.8% of the variation of phobic anxiety, and 18.8% of the variation of paranoia ideation.

**Table 3:** The relationship between the mean value and age of each factor in the SCL-90 scale of athletes.

Dimension	With controls	
	β	*R^2^*
Somatic complaints	0.219	0.048
Obsessive compulsive	0.388	0.150
Interpersonal sensitivity	0.397	0.157
Depression	0.506^**^	0.256
Anxiety	0.372	0.138
Hostility	0.522^**^	0.272
Phobic anxiety	0.488^**^	0.238
Paranoid ideation	0.434^*^	0.188
Psychoticism	0.361	0.130

*p < 0.05, **p < 0.01.

### 3.2 The change of athletes’ mental health with age

The results in Table 3 indicate that the three factor scores of depression, hostility, and terror have increased over the years. The factor weighting values of 2023 and 1995 are brought into the publicity to calculate the interpretation rate and effect size, as shown in Table 4.

**Table 4:** The change of mental health of Chinese athletes.

Dimension	*M_1995_*	*M*_2023_	*M*_变化_	*SD*	*d*	*r^2^*
Depression	1.27	1.67	0.4	0.54	0.74	0.35
Hostility	1.39	1.67	0.28	0.55	0.51	0.25
Phobic anxiety	1.10	1.52	0.42	0.44	0.95	0.43

From Table 4, it can be seen that from 1995 to 2023, the three factors in the SCL-90 scale of Chinese athletes all show an upward trend, with depression increasing by 0.4 points, hostility increasing by 0.28 points, and phobic anxiety increasing by 0.42 points. According to the judgment of the effect size, the effect size between 0.2 and 0.5 is small effect. The medium effect is greater than 0.5–0.8, and the hostile is “medium effect.” Greater than 0.8 is a large effect, and depression factor and phobic anxiety are “large effects.” All of the above indicate that from 1995 to 2023, the mental health of Chinese athletes gradually deteriorates in depression, hostility and phobic anxiety, and the change speed is relatively fast, showing a large degree.

### 3.3 Differences in mental health of male and female

In this part, the correlation between the mean value of various factors of mental health of athletes of different genders and the age was statistically analyzed. It can be seen that the somatic complaints, obsessive compulsive, hostility are positively correlated, but they are not statistically significant. Among female athletes, interpersonal sensitivity and paranoid ideation paranoia were negatively correlated with age, while the other factors were positively correlated. Among the depression factors, age could explain 67.2% of the variation, and among the psychoticism, age could explain 48.9% of the variation, indicating that the scores of depression and psychoticism psychosis of female athletes were gradually increasing with the increase of age. It can also be seen that the change of depression in female athletes is the main cause of the change of depression factors in the mental health of the whole athlete group (Table 5).

**Table 5:** Correlation between the mean value of SCL-90 factors and the age of different gender athletes.

Dimension	Male (*N =* 1,148)		Female (*N =* 1,032)	
	*β*	*R* ^2^	*β*	*R* ^2^
Somatic complaints	−0.243	0.059	0.407	0.166
Obsessive compulsive	−0.084	0.007	0.647	0.418
Interpersonal sensitivity	0.001	0.000	−0.029	0.001
Depression	0.330	0.109	0.819^**^	0.672
Anxiety	0.524	0.129	0.155	0.024
Hostility	−0.337	0.114	0.646	0.417
Phobic anxiety	0.033	0.001	0.479	0.230
Paranoid ideation	0.158	0.025	−0.150	0.022
Psychoticism	0.036	0.001	0.699^*^	0.489

*p < 0.05, **p < 0.01.

### 3.4 The difference of mental health of different types of athletes

This part makes a statistical analysis of the correlation between the mean value and age of each factor of mental health of different types of athletes. Each article reports the types of athletes, which can be divided into two types: professional athletes and college athletes. The relationship between the scores of each factor of the SCL-90 scale and the age of different types of athletes is shown in Table 6.

**Table 6:** Correlation between the mean value of each factor of SCL-90 and the age of different types of athletes.

Dimension	professional athlete (*N =* 1,482)		college athletes (*N =* 2,745)	
	*β*	*R* ^2^	*β*	*R* ^2^
Somatic complaints	0.223	0.05	0.474	0.225
Obsessive compulsive	0.344	0.119	0.516	0.267
Interpersonal sensitivity	0.214	0.046	0.510	0.260
Depression	0.324	0.105	0.672^**^	0.451
Anxiety	0.219	0.048	0.622^**^	0.386
Hostility	0.542^*^	0.294	0.566^*^	0.320
Phobic anxiety	0.297	0.088	0.681^**^	0.463
Paranoid ideation	0.230	0.053	0.703^**^	0.494
Psychoticism	0.286	0.082	0.542^*^	0.294

*p < 0.05, **p < 0.01.

Overall, different types of mental health status over the years have obvious differences. The scores of mental health factors of professional athletes and college athletes increased gradually with the change of years, and the hostility of professional athletes showed an upward trend with the change of years, the increase rate was 29.4%; The factors of depression, anxiety, hostility, phobic anxiety, paranoid ideation, psychoticism in college athletes were on the rise with the change of years, and the increase rate was between 29.4 and 49.4%. Depression, phobic anxiety, paranoid ideation and hostility change of college athletes and hostile change of professional athletes are the main causes of the change of depression, hostility, fear and paranoia of athletes.

## 4 Discussion

### 4.1 Changes of athletes’ mental health level

In order to explore the change trend of the overall mental health status of Chinese athletes in recent 28 years, this study used cross-sectional historical meta-analysis to explore the relationship between SCL-90 scale score and age. The results showed that the scores of the 9 psychological problems in the SCL-90 scale increased gradually with the age, among which depression, hostility, phobic anxiety and paranoid ideation were the most significant.

This is consistent with previous studies showing an increased risk of mental health problems among athletes (Sundgot-Borgen et al., 2004; Baum, 2005). A national survey of elite athletes in Australia found that almost half of the respondents had a mental health problem, with a prevalence similar to that of the community group (Gulliver et al., 2015).Recent studies have shown that retired elite athletes have a particularly high risk of mental illness (Gouttebarge et al., 2016).Foreign countries pay close attention to the mental health of athletes, especially elite athletes, but the overall research is relatively small at present. However, the view that elite athletes lack mental health has been paid more and more attention by sports medicine experts (Wolanin et al., 2015). Although much has been done to promote and disseminate the improvement of athletes’ mental health, as well as the prevention, identification and early treatment of mental health problems, it is hoped that the relevant sports bodies will continue their efforts to minimize the extent of mental illness in athletes (Reardon et al., 2010). If athletes do not have timely access to psycho-mental health care, or do not see this as important themselves, then this will have an impact on them to have psychological problems (Hamer et al., 2009). Almost all studies have indicated that physical exercise has a positive impact on mental health (Daley, 2008), but some studies have found that intense physical exercise by elite athletes is detrimental to mental health and will excessively increase anxiety, depression symptoms, injuries and mental exhaustion (Peluso et al., 2005). Many domestic research results show that the mental health of Chinese athletes is not optimistic, and the scores of many indicators of mental health level are higher than the national norm (Niu et al., 2009; Tan et al., 2014).The emergence of this situation is influenced by many factors, especially the social macro environment. Since China successfully held the Beijing Olympic Games in 2008, competitive sports have been booming, and with it comes social expectations for athletes, which makes athletes pay more attention to performance. The special status of athletes also leaves them with less time and experience to devote to their studies, and when it comes to college, they have to pay more than non-athletes to get into college. The common problem faced by professional athletes is the problem after retirement. The retirement of athletes has always been one of the main problems plaguing the development of competitive sports in China. In addition, there are other studies that show that the national team athletes have a serious lack of learning of cultural lessons, and 48% of the surveyed people have not taken cultural lessons in the latest year (Xu et al. 2011; Zhou et al., 2012). The management system and mode within the sports system also directly affect the future development of retired athletes. Due to the lag of system reform, the mobility, development and re-employment of athletes are objectively affected (Zhou et al., 2012).

### 4.2 Changes of mental health level of athletes of different genders

This study found that depression and psychoticism scores of female athletes gradually increased with the growth of years, and such changes were the main reason for the change of depression factors in the mental health of the entire athlete group, while there was no significant change in male athletes. This is consistent with the results of some studies, which have found that vulnerability is related to depressive symptoms (Yamaguchi et al., 2018), and the vulnerability score of women is significantly higher than that of men (Yamaguchi et al., 2019). Female athletes have more symptoms of depression and suspicion than male athletes (Zhou et al., 2005).

From an individual perspective, female athletes face a greater variety of sources of pressure compared to male athletes (Pascoe et al., 2022). These sources include pressures related to family planning, motherhood, caregiving responsibilities (Moesch et al., 2012), media scrutiny (Wensing et al. 2003; Biscomb et al. 2019), and the prevalence of violence against women in the sports industry (Donegan, 2012). Additionally, inequalities in areas such as compensation, employment opportunities, and societal roles contribute to the stress experienced by female athletes (Pascoe et al., 2022). If these pressures are not actively addressed, they can lead to mental health issues. Furthermore, considering traditional biomedical differences (Zhou et al., 2005), women exhibit greater variations in hormone systems throughout their lifecycles compared to men (Seaman, 1997), potentially increasing the risk of factors like depression and mental illness among female athletes.

From the perspective of cultural causes, the decline of women’s mental health is due to the double pressure from life and work, especially the conflict of multiple social roles and the squeeze of gender culture (Ye, 2010). Campbell et al. (2020) believes that in countries with greater gender equality, women face a double burden of balancing economic income and political participation while also adhering to traditional female responsibilities and norms. For example, the pressure on women to succeed in highly competitive environments, while also having to navigate traditional gender roles such as childbirth, may exacerbate stress levels and increase the risk of mental health issues among female athletes.

Although domestic sports institutions provide mental health education for athletes with different levels of needs by inviting psychological experts to carry out training under the team and set up basic psychological education courses, at present, professionals with clinical psychological counseling experience and familiar with elite sports are very scarce, which greatly restricts the development of mental health of athletes in China. Therefore, in order to prevent the spread of female athletes’ psychological problems and improve their mental health as soon as possible, it is also necessary to integrate various social forces and provide a more equal and harmonious social, cultural and institutional environment.

### 4.3 Changes of mental health level of different types of athletes

This study found that the scores of depression, anxiety, hostility, phobic anxiety, paranoid ideation, psychoticism of college athletes were significantly increased year by year, and the scores of hostility factors of professional athletes were significantly increased year by year. It has become an indisputable fact that there are certain problems in the mental health of college athletes. Studies have found that the rate of college athletes and club members seeking treatment from college mental health organizations is relatively low, and 46.4% of college athletes show symptoms of depression, eating disorders and anxiety disorders (Gulliver et al., 2015). Alcohol abuse is more common among college athletes than non-athletes (Ford, 2009). Studies have shown that athletes with high education have more personal, career, academic and family problems, and their mental health status is relatively poor (Chen, 2001).

On the one hand, such a result may be related to the dual pressure of college athletes who need to balance academic work and performance. College athletes have not only academic tasks but also competition tasks, and long-term conflicts in academic training will inevitably lead to certain pressure and negative emotions of college athletes. On the other hand, it may be related to the stage of college athletes. Just entering college is a key period of self-awareness cultivation, but it may be negligent management, resulting in college athletes not establishing a correct and positive self-awareness.

### 4.4 Limitations

The present study has the following limitations. First, there are other instruments to test mental health besides the SCL-90, such as the PHI (Zhou et al., 2009; Ma et al., 2015). Moreover, this study is the reliance on the SCL-90 tool for assessing mental health factors among athletes. While the SCL-90 is a widely used instrument, its effectiveness in capturing the full spectrum of mental health issues in athletes may be subject to interpretation and measurement bias. Future research could further explore the underlying mechanisms of athletes’ mental health status by comparing the results of cross-temporal meta-analyses using other questionnaires with the current results. Second, although the current study examined the trend of mental health level of Chinese athletes and provided possible reasons for this trend, it did not further reveal the specific influencing mechanisms. Therefore, future studies should adopt a longitudinal design and corresponding statistical analyses to test the relevant influencing factors. At Last, the longitudinal meta-analysis approach employed in this study, spanning different time periods and locations, introduces potential biases related to variations in data collection methods, cultural factors, and evolving societal norms. These factors could impact the generalizability and robustness of the findings.

## 5 Conclusion

This study use a cross-temporal meta-analysis to explore the changes in the mental health level of Chinese athletes, and found that the mental health level of Chinese athletes has been on an upward trend over the past 28 years. This phenomenon may be related to the implementation of national sports policies, retirement resettlement, adverse perceptions such as gold medal supremacy, and the provision of mental health education. In addition, Chinese female athletes are at higher risk of depression and psychoticism, while the mental health mental health level of college athletes deserves more attention.

## Data availability statement

The original contributions presented in the study are included in the article/supplementary material, further inquiries can be directed to the corresponding authors.

## Author contributions

YX: Data curation, Investigation, Methodology, Writing – original draft, Writing – review & editing. FL: Data curation, Funding acquisition, Supervision, Writing – original draft. JY: Conceptualization, Methodology, Project administration, Resources, Writing – review & editing.
